# Spatial heterogeneity and Immune infiltration of cellular lysosomal pathways reveals a new blueprint for tumor heterogeneity in esophageal cancer

**DOI:** 10.3389/fendo.2023.1138457

**Published:** 2023-04-05

**Authors:** Jinxing Wei, XiaoMing Wu, Shuohao Wang, Siqing Liu, Xia Gao

**Affiliations:** ^1^ Henan Provincial Chest Hospital, Zhengzhou University, Zhengzhou, China; ^2^ Department of Broad Discipline of Computing, The Hong Kong Polytechnic University, Hung Hom, Kowloon, Hong Kong SAR, China; ^3^ The Second Clinical Medical College, Tianjin Medical University, Heping, Tianjin, China

**Keywords:** lysosomes, esophageal squamous cell carcinoma, cellular autophagy, single-gene sequencing, immune infiltration

## Abstract

**Background:**

Esophageal squamous cell carcinoma (ESCC) is a common Malignant tumor of digestive tract which have a potential association with lysosomal pathway. The purpose of this study was to explore the correlation between lysosome pathway and immune infiltration of ESCC.

**Methods:**

The cell type annotation of ESCC patients and the distribution of their gene markers were analyzed by single cell data. They were also grouped according to the expression of lysosomal pathways. Gene set variation analysis (GSVA) enriched pathway scoring, Cellchat cell communication was performed to demonstrate the tumour-associated pathway scores and interactions of different cell populations. Relevant differential genes were screened, prognostic risk markers were constructed and direct associations of lysosomal pathway-related gene risk scores with immune infiltration and tumour treatment drug sensitivity were assessed by algorithms. In cellular experiments, qPCR and flow cytometry were used to assess the role of the lysosomal pathway gene-MT1X on tumour cell development.

**Results:**

ESCC single cell data were annotated into 7 Cluster clusters by t-sne downscaling analysis. Cellchat analysis revealed that the “MIF” cellular communication network is the main communication mode of the lysosomal pathway in ESCC cells. The lysosomal pathway genetic risk model was found to be significantly different from ESCC prognosis in both the training and validation groups. The lysosome pathway gene risk model was associated with treatment resistance in ESCC patients using oncopredict R package. The correlation between the expression of lysosomal-DEG and tumour immune infiltration and immune cell types by the MCPcounter method. Cellular assays showed that the lysosomal pathway gene MT1X was less expressed in oesophageal cancer cells than in normal oesophageal epithelial cells. Knockdown of MT1X significantly promoted the growth rate of oesophageal cancer cells.

**Conclusion:**

Based on the single cell sequencing technology and transcriptomic analysis, we confirmed that there is a close association between the lysosomal pathway and the immune infiltration and treatment sensitivity of ESCC, which may be a potential target for a new direction of ESCC therapy.

## Introduction

Esophageal cancer is one of the more aggressive malignancies (1). Epidemiological studies (2, 3) have shown that esophageal squamous cell carcinoma (ESCC) accounts for about 90% of esophageal cancers and esophageal adeno-carcinoma (EAC) accounts for only 10%. ESCC is therefore the main type of oesophageal cancer and has a high morbidity and mortality rate among patients with oesophageal cancer, with a 5-year survival rate of less than 20% (4). The poor prognosis is associated with difficulties in early diagnosis, frequent metastases and reduced therapeutic sensitivity (5). Therefore, there is an urgent need to develop new diagnostic, therapeutic and prognostic assessment strategies to improve the overall survival of EC patients. At the same time, further in-depth studies on the altered immune infiltration microenvironment and molecular pathway amount in esophageal cancer, combined with information on tumour heterogeneity, are needed to dissect the intrinsic features of ESCC from a molecular perspective (6–8). It is an important factor to promote the development of new clinical ESCC therapies and innovative esophageal cancer treatment strategies.

Autophagy is a process by which self-damaged organelles and proteins are separated in autophagic vacuoles (AVs) and transported to lysosomes for catabolism (9). The nucleation of AVs is mediated by the mammalian target of rapamycin complex 1 (mTORC1) and adenosine monophosphate activated protein kinase (AMPK); the extension and maturation of AVs is regulated by autophagy related gene (ATG) and phosphoinositide 3-kinase (PI3Ks) (10). Recent studies have shown that autophagy regulates tumour cell growth both as a promoter and an inhibitor, and that targeting autophagy may influence the efficacy of anti-tumour therapy (11, 12). However, the important functions of autophagy and lysosomal pathways in the development of ESCC have not yet been reported and systematically summarized (13). As esophageal cancer is a tumour type with high tumour antigenicity and cross-over effects of immunotherapy, we suggest that the lysosomal pathway and cellular autophagy have important potential in the exploration of new therapies and assessment of immune infiltration in ESCC.

Herein, we investigated the specific roles of lysosomal-related pathways and cellular autophagy in the development of oesophageal carcinogenesis and invasive metastasis in tumour samples from ESCC patients, and further determined the spatial specificity of the distribution of related genes and cellular pathways in ESCC cells by means of single-cell sequencing and spatial transcriptomic analysis. The results showed that subpopulations of tumour cells with different lysosomal pathway-associated gene profiles appeared heterogeneously distributed between and within tumour foci. This suggests that aberrant distribution of the lysosomal pathway may determine poor prognosis and immune tolerance in ESCC patients. In addition, we assessed the specific relationship between the lysosomal pathway and related genes and immune infiltration in ESCC, and constructed subgroups to assess their impact on drug sensitivity. These findings provide new insights into the spatial characteristics, complex ecosystem and biological behaviour of ESCC clones, and provide new insights into individualized treatment of ESCC.

## Materials and methods

### Methods

#### Data acquisition

The GEO public gene expression data and full clinical annotation were searched, and this study included bulk RNA-seq from patients in the GSE53624 cohort (including 117 oesophageal squamous carcinomas), GSE53622 cohort (including 60 oesophageal squamous carcinomas), and single-cell scRNA-seq data from five patients with oesophageal squamous carcinomas in the GSE188900 cohort for further analysis. The RNA-Seq data were corrected for batch effects using the R package “sva” (version 3.44.0). Each included patient contained complete matching clinical data such as age, gender, tumour stage, TNM stage, survival status, etc. The inclusion and exclusion criteria for this study were as follows. Inclusion criteria: (1) follow-up time of at least 30 d; (2) primary oesophageal tumour; (3) inclusion of data related to mRNA, lncRNA and miRNA gene expression levels; (4) complete personal basic information, pathological information and follow-up information of the patient. Exclusion criteria: (1) secondary oesophageal tumours; (2) concurrent primary tumours from other sites.

#### Lysosome-related pathway gene acquisition and single-cell data pre-processing

Lysosomal-related pathways were included in this study from the MSigDB database: including CCDC115, CLN3, DPP7, GBA, LAMP2, LAPTM4B, LDLR, LRP1, LRP2, MARCHF2, MFSD8, MGAT3, TMEM106B, TMEM199, TPP1, VPS13A, and VPS35, a total of 17 genes. The expression of lysosomal pathway-related genes was collected and analysed in the GSE53624 cohort and the GSE188900 cohort, respectively. Single-cell analysis of the scRNA-seq data from GSE188900 was performed on five oesophageal squamous carcinoma samples. t-distributed stochastic neighbor embedding (t-SNE) is a machine learning algorithm for dimensionality reduction, which is very suitable for visualizing high-dimensional data down to two-dimension or three-dimension. The AUCell R package was used to determine the lysosomal pathway activity of each cell line in seven cell populations, which were divided into two groups, Lysosome-high and Lysosome-low, according to the median AUC score. Cell population grouping was performed by post-processing of single cell sequencing data acquisition and downscaling analysis. This included Fibroblasts, Myeloid and Endothelial cell groups as well as the remaining cell types. GSVA enrichment pathway scores were collected and calculated for both Lysosome-high and Lysosome-low groups using 50 Hallmark datasets.

#### Cellular communication and tumour-associated pathway analysis of single-cell sequencing data from ESCC samples

After pre-processing and downscaling analysis of single cell scRNA-seq data from five patients with esophageal squamous carcinoma from GSE188900, CellChat scores were calculated for the seven cell populations of the downscaled subgroups, demonstrating the cellular communication of each cell population, as well as the relationship between the Lysosome-high group dominated by Fibroblasts, Myeloid and CellChat constructed a database of cellular interactions containing 2021 ligand-receptor pairs. CellChat can be used to quantify intercellular communication networks from single cell transcriptomic data, to resolve the major input and output signals of each cell type, and to suggest how each cell type and multiple signalling pathways operate in concert. The Macrophage migration inhibitory factor (MIF) signalling pathway is the secretory signalling pathway with the highest probability of communication in ESCC cells, and we demonstrate the cellular communication network of the “MIF” pathway. We also calculated PROGENy scores to show the scores of tumour-associated pathways in different cell populations.

### Gene enrichment analysis for differential genes in lysosome-associated pathways

The R package “LIMMA” (version 3.48.3) was applied to compare the Lysosome-high and Lysosome-low groups. LMFIT and EBayes functions were used to ensure accuracy. Differentially expressed genes (DEGs) were screened with adjusted P values < 0.05 and absolute values of logFC > 0.585. GO functional enrichment analysis and KEGG metabolic pathway enrichment analysis were performed on DEGs and core genes using the R packages clusterProfiler, org.Hs.eg.db, DOSE, enrichplot, colourspace, etc. GO enrichment analysis can annotate genes with significant differences at three levels: cellular component, molecular function and biological process. The cellular component describes the location of the differential gene, such as the cytoplasm, nucleus or mitochondria. Molecular function describes the function of the differential gene at the molecular biological level. Biological processes mainly describe the biological processes in which the differential genes participate, such as regulation of cell proliferation, cell development and cell migration. The present study can summarise the large number of differential genes at the cellular component, molecular function and biological process levels, reflecting the macroscopic association of ESCC with the lysosomal pathway.

### Acquisition of survival-related genes from lysosomal pathways and construction of prognostic models

The differentially expressed genes (DEGs) obtained in the previous step were subjected to univariate Cox regression analysis based on the tinyarray package, with p-value < 0.05 as the screening criterion, and a total of 117 genes were selected. The GSE53624 cohort was used as a training cohort to select genes with prognostic significance from the 117 genes and construct a prognostic model based on Randomforest random forest. After calculating the median for the risk score, this median was distinguished between the High-risk and Low-risk groups by using Kaplan-lysosome pathway-related genes to intervene in the possible mechanisms of ESCC survival. For having significant prognostic differences. And independent external validation was performed by applying the cohort GSE53622 of 60 patients with esophageal squamous carcinoma.

### Scoring of immune infiltration levels and evaluation of treatment sensitivity for markers of lysosomal-related pathways

To further clarify the relevance of lysosomal pathway-related genes to immune infiltration and ESCC drug resistance. We assessed the level of immune infiltration by 3 algorithms, Cibersort, ssGSEA and MCP-Counter. Based on the expression matrix, Cibersort used a deconvolution algorithm to assess the composition and abundance of immune cells in mixed cells. Based on the expression matrix and the immune cell Marker gene set, ssGSEA calculates enrichment scores for single samples and gene set pairs to determine the level of immune infiltration. ssGSEA uses transcriptomic data to quantify the abundance of immune cells and stromal cells. The TIDE algorithm predicts tumour response to immunotherapy, correlating expression matrices and T cell dysfunction in tumours with high Cytotoxic T lymphocyte (CTL) expression, predicting high correlation in patients with no response to immunotherapy. In CTL low expression tumours, the expression matrix and T-cell rejection characteristics of tumour patients were correlated to predict highly relevant patients as non-responders to immunotherapy. In addition, immune scores and tumour purity were calculated for each sample by the ESTIMATE algorithm.

In addition, we tested the therapeutic sensitivity of lysosomal-related pathway markers to a variety of chemotherapeutic agents to further explore the clinical reality of the association between the lysosomal pathway and ESCC drug resistance. The R package “pRRophetic” was used to predict drug sensitivity based on the Cancer Genome Project (CGP) database. Similarly, drug sensitivity scores were calculated by the R package “oncoPredict” based on the Genomics of Drug Sensitivity in Cancer (GDSC) database.

### Cell culture and flow cytometry validation

Human oesophageal cancer cells Eca-109 and normal oesophageal epithelial cells HET-1A were purchased from Shanghai Cell Bank, Chinese Academy of Sciences; RPMI-1640 was purchased from Gibco; fetal bovine serum was purchased from Thermo Fisher. The frozen Eca-109 cells and HET-1A cells were recovered and inoculated into RPMI-1640 medium containing 10% fetal bovine serum and incubated at 37°C in a 5% CO2 incubator. Differences in the expression of lysosome-related genes between ESCC cells and normal cells were measured and analysed. Independent control groups were set up to construct cell lines with knockdown MT1X gene and two replicate groups were set up. The cells were collected, centrifuged at 400 × g for 5 min, the supernatant was discarded, washed twice with pre-chilled PBS, added with pre-chilled 75% ethanol and fixed in a refrigerator at -20°C for more than 24 h. The cells were centrifuged at 700 × g for 5 min, and the supernatant was discarded. The cells were incubated for 10 min at 4°C, protected from light. The cell cycle was measured on a flow cytometer. The cells were transfected 1 day prior to transfection at a final concentration of 100 nmol/L. After 8 h of transfection, the cells were replaced with complete culture medium. After 48 h of transfection, total RNA was extracted and cDNA was synthesized by MMLV reverse transcriptase, and the interference efficiency was measured by real-time quantitative PCR using β-actin as internal reference. The cycling conditions for MT1X and β-actin were as follows: 95°C for 5 min, 95°C for 10 s, 61°C for 15 s and 85°C for 5 s. A total of 30 cycles were performed. The MT1X gene value was divided by the β-actin gene value to calculate the expression of the sample.

## Results

### Initial visualization and distribution analysis of single-cell sequencing of ESCC cells

**Figure 1:**
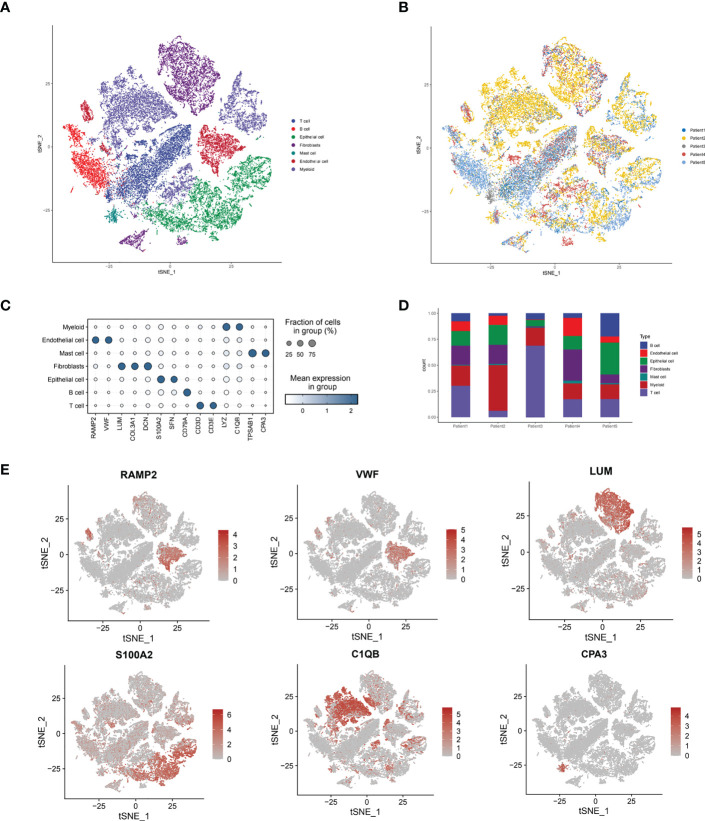
Sample immune cell type analysis and lysosomal pathway gene distribution by single cell sequencing of ESCC cells. **(A)** t-SNE diagram showing the distribution of 7 major cell types in ESCC cells: Tcell.B cell,Epithelial cell, Fibroblasts, Mast cell, Endothelial cell and Myeloid; **(B)** t-SNE diagram showing the source of the cell samples, each colour indicates one sample, a total of 5 patients are included; **(C)** bubble diagram of the major genes and groups expressed in the 7 cell types obtained from single cell sequencing, the results show that in Myeloid cells, LYZ and C1QB genes are highly expressed; in Endothelial cells, RAMP2 and VWF have high expression. LYZ and C1QB genes are highly expressed; in Endothelial cells, RAMP2 and VWF possess high expression; **(D)** Cascade bar graphs further visualize the specific type number share of the 7 immune cell types in ESCC patients, with Myeloid cells, T cells, Fibroblasts more annotated and other cell types less abundant; **(E)** t SNE plots show the expression of typical marker genes for the different cell types.

By collecting single-cell scRNA-seq data from the GSE188900 cohort of five patients with esophageal squamous carcinoma, we mapped a comprehensive multi-locus single-cell transcriptome profile of ESCC. After expression normalization, cells were subsequently classified into coherent transcriptional clusters (Clusters) using a graph-based clustering approach. The cells were divided into Clusters by t-sne descending analysis. We annotated the cells in clusters and grouped the Clusters into seven main categories, namely: Tcell.B cell, Epithelial cell, Fibroblasts, Mast cell, Endothelial cell and Myeloid (**Figure 1A**), and annotated the cells according to their sample origin. The distribution of cells shows that the different types of immune cells have distinctly different subspatial locations in the ESCC cells. The Fibroblasts are mainly located on the upper side of the ESCC, with Myeloid and T-cells in close proximity; the B-cells and Endothelial cells are located closer together, mainly on the left side of the axis; the Mast cells and Endothelial cells are mainly located in the The other main distribution area of Mast cells and Endothelial cells is located in the lower right corner of the axis. We can further see that Endothelial cells, T cells and B cells are the main cell types annotated in the ESCC single cell sequencing. Immediately afterwards, we annotated the cells according to their sample origin. As seen in **Figure 1B**, the spatial distribution of the samples from the five ESCC patients is also characteristic: Patient 1, Patient 2 and Patient 5 samples yielded more cell annotations, occupying 70-80% of the entire space. The annotation information obtained for Patient 2’s sample was mainly located in the upper half of the space, while Patient 4’s annotation information was more sporadic and less obtained. Having obtained the spatial distribution characteristics of ESCC immune cell types, we wanted to further understand the association of immune cell types with lysosomal pathway-related genes and the positions occupied in the grouping (**Figure 1C**). In Myeloid cells, LYZ and C1QB genes were highly expressed; in Endothelial cells, RAMP2 and VWF were highly expressed; in Mast cells, TPSAB1 and CPA3 were associated; and in B cells, CD79A was consistently expressed. The expression of T cells and Epithelial cells was less consistent with CD79A. In **Figure 1D**, we further visualise the cell type representation of several patients by means of cascading bar charts. Patient 1 and patient 2 had a relatively similar distribution of cells, with Myeloid cells and T cells predominating. In contrast, for the overall five patients, all basically showed a higher annotation of Myeloid cells, T cells, Fibroblasts and a lower content of other cell types. We mapped t-SNE based on specific expressed genes in different types of clusters and found typical genetic markers used to identify cell types, the results of which are shown in **Figure 1E**. In Endothelial cells, RAMP2 and VWF possessed high expression; LUM was distributed in Fibroblasts cells; S1002 was distributed in Epithelial cells, while C1QB and CAP3 were located in Myeloid and Mast cells, respectively.

### Cell grouping and the exploration of signaling pathways

**Figure 2:**
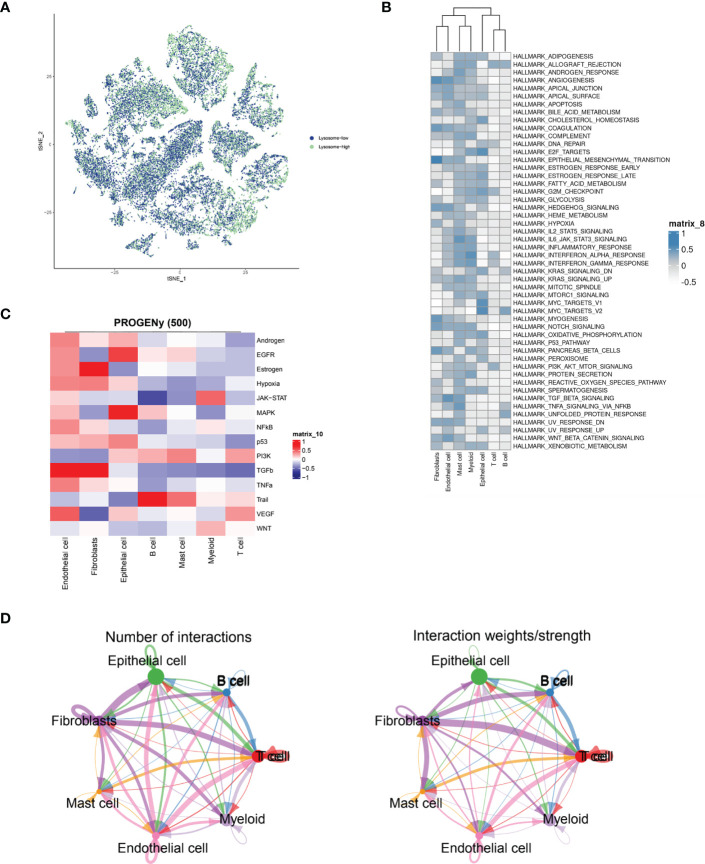
ESCC immune cell types interacting with lysosomal pathway and GSVA enrichment analysis. **(A)** t-SNE plots showing the expression of lysosomal pathway-related genes in seven major cell types in ESCC cells, divided into two groups, lysosome-high and lysosome-low, based on colour; **(B)** GSVA enrichment pathway scores were calculated for the Lysosome-high and Lysosome-low groups using 50 Hallmark data sets; **(C)** Calculation of PROGENy scores will obtain heat maps showing the scores of tumour-associated pathways for different cell populations; **(D)** Circle interaction plots showing the number and weight analysis of ineraction of Fibroblasts cells with the remaining several immune cells.

Immediately afterwards, to assess the correlation between specific immune cell types and the lysosomal pathway in ESCC patients, we applied the AUCell R package to determine the lysosomal pathway activity of each cell line (**Figure 2A**). The groups were divided into Lysosome-high and Lysosome-low according to the median AUC score value. It can be seen that Fibroblasts, Myeloid and Endothelial cells are predominantly in the Lysosome-high group and the remaining cells are predominantly in the Lysosome-low group. This suggests that several ESCC metabolic pathways and cellular synthesis processes, namely fibrosis, endothelial formation, and myeloid neoblast processes, may be closely related to the lysosomal pathway of ESCC, pending our further study in the future. In **Figure 2B**, GSVA enrichment pathway scores were calculated for both Lysosome-high and Lysosome-low groups using 50 Hallmark data sets. For GSVA pathway enrichment analysis, the average gene expression for each cell type was used as input data using the GSVA package. Results show. In Fibroblasts cells, ANGIOGENESIS, EPITHELIAL_MESENCHYMAL_TRANSITION, MYOGENESIS and Notch were predominantly expressed; in Endothelial cells, WNT_BETA_CATENIN and TGF_beta expression; Mast cells were associated with IL6_JAK_STAT3_SIGNALING and TNFα; Epithelial cells were mainly associated with E2F_targets and MYC_TARGETS_V1 and MYC_TARGETS_V2; while T cells and B cells lacked significant pathway correlation. Further, we calculated PROGENy scores to demonstrate the scores of tumour-associated pathways for different cell populations (**Figure 2C**). As can be seen, Endothelial cells are associated with a variety of tumour pathways, including Androgen, EGFR, Estrogen, Hypoxia, JAK-STAT, MAPK, NFKB and TGFb. Fibroblasts are highly correlated with Estrogen pathway expression. cells were significantly associated with MAPK and EGF pathways. For other cell types, there was less agreement with tumour-related pathways. In **Figure 2D**, the cellular communication of each cell population is shown by calculating the CellChat score, an open source R package (http://github.com/sqjin/CellChat) that can use scRNA-seq data to infer, visualise and analyse intercellular communication. For cell types with high lysosomes, Fibroblasts had more interactions with Epithelial cells and T cells; while communication between Fibroblasts and Epithelial cells, T cells, B cells and Mast cells was highly weighted in the overall ESCC cell tumour expression composition.

### The communication networks and interactions analysis between cells based on ESCC single-cell sequencing data

**Figure 3:**
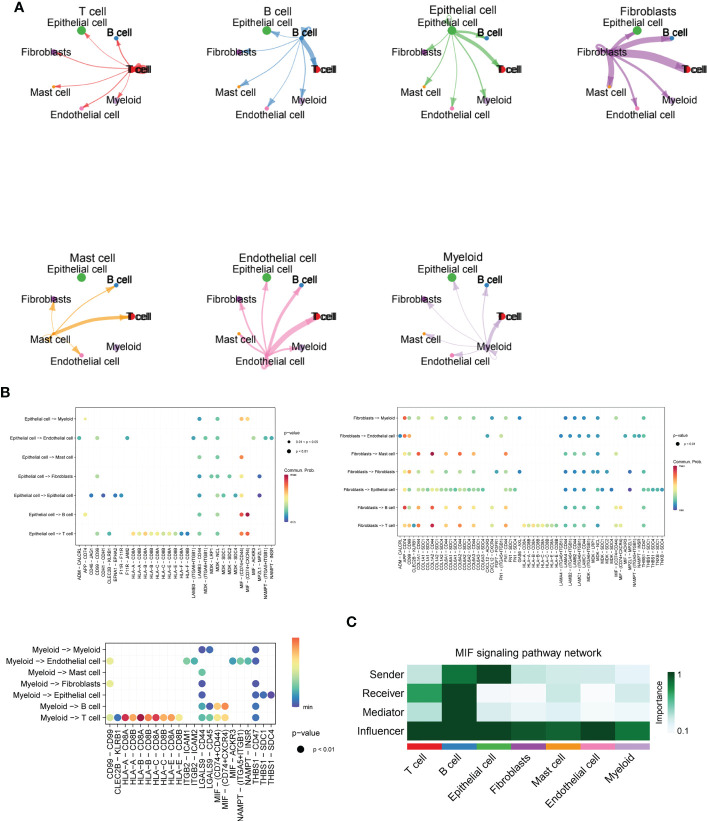
Cellchat and MIF signalling communication between immune cell types in ESCC. **(A)** Cross-correlation map of immune cell types obtained from single-cell sequencing data of ESCC patients, including T cells, B cells, Fibroblasts, Mast cells, Endothelial cells, and Myeloid cells; **(B)** Bubble plots further quantitatively visualize the major intercellular links of several lysosomal-high cells as well as mediating cytokines, including Fibroblasts, Myeloid, and Epithelial cells.**(C)**. Heat map demonstrating the prominence of each cell subpopulation as a major sender, receiver, mediator and influencer at the centre of the MIF secretory signalling communication network. B cells and EPITHELIAL cells play the role of major MIF signal senders. B cells are also important Receivers and Mediators, while throughout the MIF signalling pathway, T cells, B cells as well as Epithelial cells played a major Influencer role in the overall MIF signalling pathway.

In **Figure 3A**, we delve further into the interactions of immune cell types obtained from single cell sequencing data from ESCC patients. As seen in the images, T cells interacted more uniformly with all types of cells, while B cells sent more signals to T cells. Epithelial cells interacted more with T cells and B cells. Fibroblasts, as the main cell type that sends signals, send more signals to T cells, B cells and EPITHELIAL cells, while Mast cells, Endothelial cells and Myeloid cells have more interactions with T cells. This suggests that T cells may function as the primary recipient of intercellular communication signals in ESCC patients. Immediately afterwards, we further quantitatively visualized the major intercellular links as well as mediating cytokines of several lysosomal-high cells by means of bubble plots. In **Figure 3B**, we found that Fibroblasts interaction with Myeloid is mainly mediated by APP-CD74, interaction with Endothelial *via* CD99 and CD74, transmission of MastCell signals *via* COL1A1 - CD44, COL6A2 - CD44, and FN1 - CD44, and their communication with B and T cells For Epithelial cells, there is more interaction and signalling to T and B cells, mediated by the MIF - (CD74+CD44) and MIF - (CD74+CXCR4) pathways. Myeloid cells, on the other hand, interact less efficiently with a variety of cell types and also mediate their immune effects by signalling to T and B cells. -A - CD8B, HLA-B - CD8A, HLA-B - CD8B, HLA-C - CD8A, HLA-C - CD8B, and HLA-E - CD8A. This shows that multiple cellular pathways can mediate the interaction of Myeloid with T cells. Subsequently, in **Figure 3C**, we show by heat map the significant extent to which each cell subpopulation plays a major role as a central sender, receiver, mediator and influencer of the MIF secretory signalling communication network. b cells and EPITHELIAL cells play the role of major MIF signalling senders. b cells are also important Receiver and Mediator, while throughout the MIF In the signalling pathway, T cells, B cells and Epithelial cells play the role of major Influencer. this parallels our knowledge of the tumour immune system, where T cells and B cells play the role of major signal receivers and enforcers.

### Lysosomal pathway-related transcriptomic risk model construction and functional linkage analysis for ESCC

**Figure 4:**
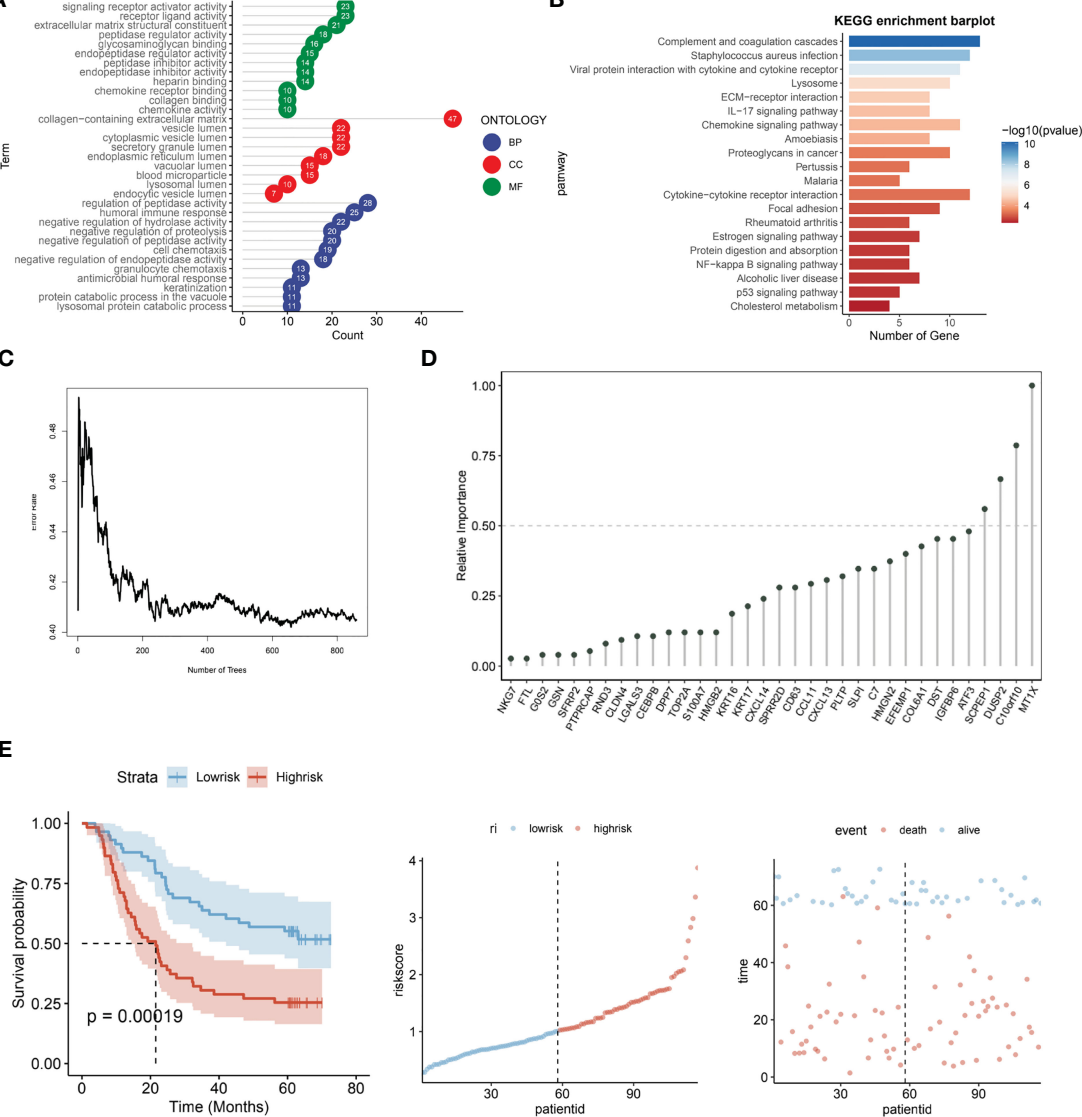
Prognostic risk modeling and functional enrichment analysis of lysosomal pathway-related transcriptomics in ESCC. **(A)** GO analysis revealed that DEG associated with lysosomal expression was mainly enriched in signaling receptor activator activity,receptor ligand activity,extracellular matrix structural constituent -peptidase regulator activity,glycosaminoglycan binding,endopeptidase regulator activity; **(B)** KEGG enrichment barplot showed that lysosomal DEG-related pathways were mainly enriched in omplement and coagulation cascades, Staphylococcus aureus infection, Viral protein interaction with cytokine and cytokine receptor enriched in; **(C)** Transcriptomic risk model construction for lysosomal-DEG by decision trees constructed from random forest; **(D)** Histogram analysis of the relative importance of lysosomal-DEG models in the final transcriptomic prognostic risk model, with MT1X possessing the highest prognostic-related importance; **(E)** Assessment of the predictive power of the constructed lysosomal pathway-related transcriptomic prognostic risk models, from left to right. Kaplan-Meire curves for the lysosome-associated prognostic risk model, distribution of risk scores, and scatter plots of prognostic survival times for patients with different risk scores.

The R package “LIMMA” (version 3.48.3) was applied to compare the Lysosome-high and Lysosome-low groups. LMFIT and EBayes functions were used to ensure accuracy. Differentially expressed genes (DEGs) were screened for adjusted P values < 0.05 and absolute values of logFC > 0.585. statistically significant lysosome-DEGs were thus obtained. and subjected to GO analysis and KEGG analysis. As shown in **Figure 4A**, the DEGs associated with lysosomal expression were mainly enriched in signaling receptor activator activity, receptor ligand activity, extracellular matrix structural constituent -peptidase regulator activity, glycosaminoglycan binding,endopeptidase regulator activity,peptidase inhibitor activity,endopeptidase inhibitor activity, heparinbinding-collagenbinding, regulation of hydrolase activity and negative regulation of proteolysis -negative regulation of peptidase activity. Further, we performed KEGG functional analysis to understand the importance of lysosomal-DEG in cell development, pathway expression and growth and development through multiple perspectives, as shown in **Figure 4B**. The results indicate that the lysosomal DEG-related pathway is mainly associated with ommunition and coagulation cascades that Staphylococcus aureus infection, Viral protein interaction with cytokine and cytokine receptor, the Lysosome, ECM-receptor interaction, IL-17 signaling pathway, Chemokine signaling pathway Amoebiasis, Proteoglycans in cancer, pathway, Pertussis, Malaria, and Cytokine-cytokine receptor interaction were related. After obtaining the relevant functional characteristics of lysosomal DEG, we selected four genes of prognostic significance from 117 genes according to the Randomforest random forest algorithm and constructed prognostic models, namely SCPEP1, DUSP2, C10orf10, and MT1X. decision tree simulations for the random forest analysis of lysosomal DEG are shown in **Figure 4C** shows. The weights occupied by the expression of different genes in the lysosomal-DEG prognostic model were further quantified in the form of dotted line plots, as seen in **Figure 4D**. MT1X was the main lysosomal-DEG gene that determined the difference in prognosis of ESCC patients, with a relative importance close to 1.0. while the relative importance of all three genes, SCPEP1, DUSP2, and C10orf10, was also higher than 0.50, significantly higher than the other lysosomal-DEG genes. In **Figure 4E**, we further analyzed the prognostic predictive power of the constructed lysosomal pathway-associated transcriptomic risk model. The results showed that the model exhibited good survival prediction performance in the GSE53624 cohort. Differentiating the High-risk and Low-risk groups by the median risk score, the two groups had significant prognostic differences. Patients in the Low-risk group in the lysosomal-DEG-related prognostic risk model had significantly better prognostic survival than those in the High-risk group (p<0.0001), while patients in the High-risk group had a significantly shorter prognostic survival time than those in the Low-risk group and were visualised in the scatter plot. This suggests a close association between lysosomal pathway-related genes and ESCC survival, perhaps related to the underlying functional characteristics of the lysosomal pathway and the specific mechanisms of immune tolerance to tumours.

### Immuno-infiltration analysis of a lysosomal pathway-associated transcriptomic risk model for ESCC

**Figure 5:**
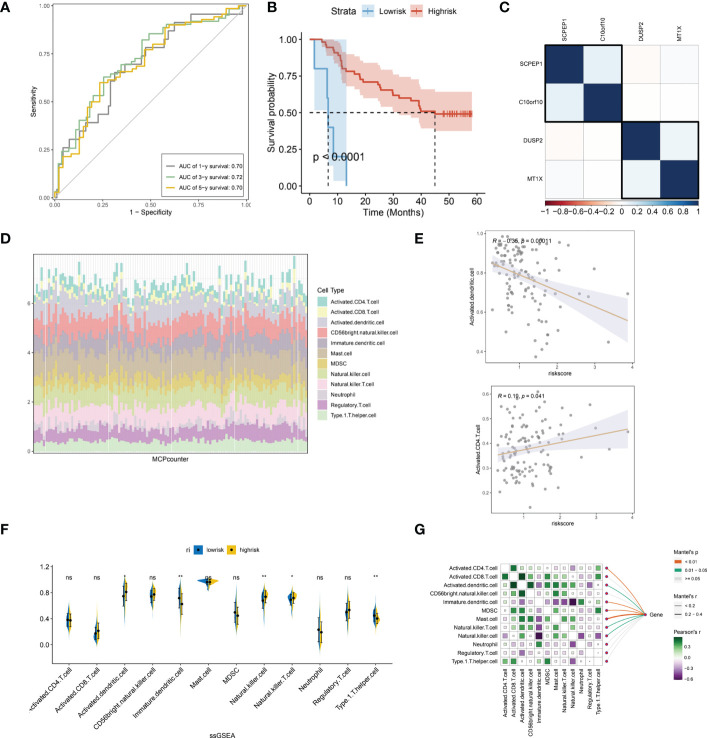
Independent validation of the lysosomal pathway-associated risk model and preliminary analysis of immune infiltration. **(A)** Prognostic prediction ROC curves for the lysosomal pathway-associated prognostic risk model ,with AUC values of 0.70, 0. 72 and 0.70 for 1-year, 3-year and 5-year, respectively; **(B)** Kaplan-Meier curves for the prognostic risk model in the GSE53622 independent validation cohort, p<0.0001. **(C)** Heat map revealing the expression correlation of the four lysosomal-DEGs that comprise the lysosomal pathway risk model, with SCPEP1 possessing a high expression correlation with C10orf10 and a significant association between DUSP2 and MT1X expression; **(D)** MCPcounter calculated histograms analyzing the potential immune cell constitutive types of risksocre; **(E)** in addition, we analysed the correlation between the expression of activated dendritic cells as well as activated CD4 T cells and risk scores; **(F)** ssGSEA analysis, revealing the enriched expression of immune cells and immune infiltration pathways between the low and high risk groups. Among them, the expression of Neutrophill, lmmature.dendritic and Th1 T cells was significantly higher in the low-risk group than in the high-risk group. In contrast, the expression of NK T cells as well as Activated.dendritic.cells was significantly higher in the high-risk group than in the low-risk group; **(G)** Heat map reveals the correlation between the expression of different immune cells and immune pathways using Pearson correlation. * means <0.05,** means <0.01, ns means >0.05.

We further evaluated the prognostic ROC curves of the lysosomal pathway-associated prognostic risk model (**Figure 5A**) with AUC values of 0.70, 0.72 and 0.70 for the 1-year, 3-year and 5-year cohorts, respectively, indicating that our constructed lysosomal risk model has good prognostic performance. In the GSE53622 cohort, we further evaluated and analysed the prognostic performance of the constructed risk model in an independent validation cohort of ESCC. In **Figure 5B**, the Kaplan-Meier curve in the validation cohort also achieved excellent prognostic prediction performance at P<0.0001. We also analysed the correlation of the expression of the four lysosomal-DEGs constituting the risk model by correlation heat map (**Figure 5C**). The results showed that SCPEP1 had a high correlation with C10orf10, suggesting that these two genes may play a similar role in the development of the lysosomal pathway. In contrast, there was a significant association between the expression of DUSP2 and MT1X. Further, we calculated the correlation and significant association between the expression of lysosomal-DEG and tumour immune infiltration and immune cell types by the MCPcounter method (**Figure 5D**). In **Figure S1D**, we showed that all types of immune cells and immune infiltrate types were highly expressed in the risk scores associated with the lysosomal pathway in ESCC patients. The expression of activated dendritic cells and activated CD4 T cells showed a high correlation with the tumour-related risk score (**Figure 5E**), with a negative correlation between the expression of activated dendritic cells and the risk score (p<0.001) and a positive correlation between the expression of activated CD4 T cells and the lysosomal pathway-related risk score (p<0.05), with a significant linear relationship. This suggests that the lysosomal pathway has a significant positive value for immune infiltration and development of tumour resistance in T cells as well as in dendritic cells. In **Figure 5F**, we further assessed the enrichment of immune cells and immune infiltrative pathways between the low-risk and high-risk groups by GSEA analysis. Among them, the expression of Neutrophill, lmmature.dendritic as well as Th1 T cells was significantly higher in the low-risk group than in the high-risk group. In contrast, the expression of NK T cells as well as Activated.dendritic.cells was significantly higher in the high-risk group than in the low-risk group. Further, our Pearson correlation in **Figure 5G** reveals the correlation between the expression of different immune cells, immune pathways. Activated CD4 T cells had a higher expression correlation with activated CD8 T cells, and activated CD8 also had a higher expression correlation with MDSK and Th1T cells. The rest of the immune cells were also more or less correlated with each other.

To further determine the specific direct association of lysosomal pathway-related risk genes with immune infiltration and immune cell secretion in ESCC, we calculated immune infiltration scores by three methods: ssGSEA, MCPcounter, and the xCell algorithm, which were visualized with box plots, heat maps, and scatter plots, respectively. **Figure S1A** shows the immune cell scores between the low-risk and high-risk groups calculated with the xCell method. The expression of CD4 Memory T cells, Macrophages, and Marcophages M1 was higher in the low-risk group than in the high-risk group; while Basophils, CLP, Epithelial, and HSC cells were less expressed in the low-risk group than in the high-risk group. In **Figure S1B**, we also applied box plot depictions to compare immune pathway scores between ESCC lysosome-associated low-risk and high-risk groups, with MEP, Monocytes, Neurons, smooth muscle, and Th2 cells being higher in the low-risk group than in the high-risk group; and Myocytes and Pericytes being more expressed in the high-risk group than in the low-risk group. In addition, we also compared the linear correlation between several immune cell types with significantly different expression and their respective risk scores, and the results are shown in **Figure S1C**. The results showed that Neutrophils, CD4 memory T cells and Macrophages M1 expression were linearly and negatively correlated with risk scores (p<0.05). In contrast, Epithelial cells as well as Myocytes corresponded to a linear positive correlation with risk score, suggesting a differential role of different cell types in the lysosomal pathway contributing to the development of ESCC (p<0.05). In addition, we analysed the interaction of genes constituting the lysosomal pathway risk model with immune infiltrating cells and pathways by correlation heat map (**Figure S1D**). the MT1X gene was mainly negatively correlated with Adipocyts, B-cellsdun, Class-switched memory T cells, HSC, Neurons expression (p<0.05), with CD8 naive T cells, MSC, NK Cells, and NKT expression. c10orf10 gene was significantly positively correlated with aDC, CD4 memory T cells, CD8 T cells, CD8 Tcm, macrophages, macrophage M1 type, and macrophage M2 type. The DUSP2 gene was significantly associated with the expression of CD4 Tem, Adipocytes, Epithelial and macrophages. For the SCPEP1 gene, there was a high correlation with macrophages and smooth muscle cells.

We also applied the MCP method to calculate the immune infiltration scores of patients in the low-risk versus high-risk groups of the lysosomal pathway and visualized them using box line plots (**Figure S2A**). Fibroblasts expression was lower in the low-risk group than in the high-risk group, while B lineage expression was higher in the low-risk group than in the high-risk group. The expression of B lineage, T cells, Neutropilis, Monocytic Lineage, Myeloid dendritic and Endothelial cells all showed a linear correlation with the risk score. The expression of B lineage, T cells, Neutropilis, Monocytic Lineage, Myeloid dendritic and Endothelial cells were linearly and negatively correlated with the corresponding risk scores, the same as those calculated by our XCell method (**Figures S2B, S2C**). In **Figure S2D** we used Pearson’s correlation to reveal the correlation between the expression of different immune cells and immune pathways in the MCPcpunter method, where T cells showed a high correlation with CD8 T cells, B lineage, Monocytic lineage and Meyloid dendritic cells, while Monocytic Lineage showed a high correlation with Monocytic Lineage was highly correlated with the expression of Endothelial cells and B lineage. Similarly, we also analysed the interaction of genes constituting a risk model for the lysosomal pathway with immune infiltrating cells and pathways by correlation heat map (**Figure S2E**). MT1X gene was mainly negatively correlated with B lineage and Cytotoxic lymphocytes expression (p<0.05), and with Endothelial and Monocytic lymphocytes expression. The C10orf10 gene was significantly positively correlated with aDC, CD4 memory T cells, CD8 T cells, CD8 Tcm, macrophages, macrophage M1 type, and macrophage M2 type. In contrast, the DUSP2 gene was significantly associated with the expression of CD4 Tem, Adipocytes, Epithelial and macrophages. For the SCPEP1 gene, there was a high correlation with macrophages and smooth muscle cells.

### Analysis of the role of ESCC lysosomal pathway risk models on tumour treatment sensitivity

**Figure 6:**
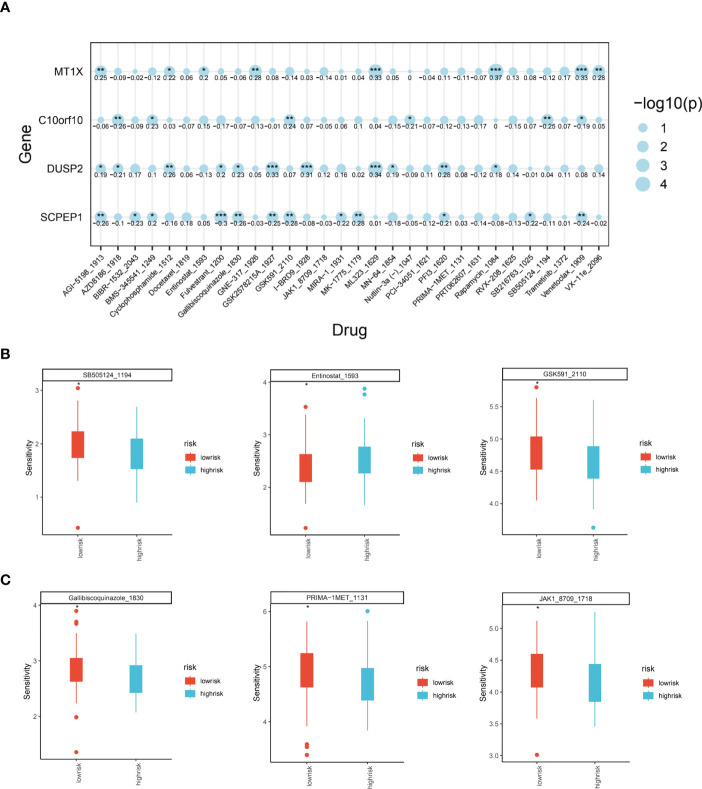
Analysis of the role of ESCC lysosomal pathway risk models on tumor treatment sensitivity. **(A)** Bubble diagram showing the association of four key genes constituting the lysosomal pathway risk model with the therapeutic sensitivity of multiple chemotherapeutic agents: MT1X was associated with AGI-5198_5913, Cyclophosphamide, ML323, Rapamycin, Venetoclax. expression of C10orf10 was significantly associated with AZD8186, GSDK591, SB505124. GSDK591, SB505124 were significantly associated. for DUSP2, it was associated with GSK2578215, I-BRD9, ML-323. For SCPEP1, treatment with Fulvetrant, MK-1775, Venetoclax_1909 had a high correlation with it. **(B)** Box plot of treatment sensitivity between low and high risk groups for lysosomal risk scores for several chemotherapeutic agents, SB505124_1194, Entinostat_1593, GSK591_2110; **(C)** Gallibiscoquinazole_1830, PRIMA-1MET_1131, JAK1_8709_ 1718 Box line plot of treatment sensitivity of these chemotherapeutic agents between low risk and high risk groups for lysosomal risk scores. * means <0.05,** means <0.01,*** means <0.001.

Through the previously constructed risk model and the relationship between immune infiltration, we have explored in depth the significant association between lysosomal pathway genes and tumour immunity and tumour cellular pathways with poorer prognosis in ESCC patients. Considering the current poor immunotherapeutic effect of ESCC and the strong immunogenicity of the tumour, we further investigated whether the lysosomal pathway gene risk model is associated with treatment resistance in ESCC patients We further investigated whether the lysosomal pathway gene risk model was associated with treatment resistance in ESCC patients. A sensitivity score was calculated for drugs in the GDSC database based on the R package “oncoPredict”. MT1X was associated with the therapeutic susceptibility of AGI-5198_5913, Cyclophosphamide, ML323, Rapamycin, Venetoclax. AZD8186, GSDK591, SB505124 was significantly associated with treatment efficacy. expression of DUSP2 in ESCC patients was associated with treatment efficacy of GSK2578215, I-BRD9, ML-323. For SCPEP1, treatment with Fulvetrant, MK-1775, Venetoclax_1909 was highly correlated with it (**Figure 6A**). In **Figures 6B, C**, we applied box-line plots to analyse the potential correlation between several key chemotherapeutic agents used for ESCC treatment and the distribution of lysosomal pathway risk models. The results show that for several chemotherapeutic agents, SB505124_1194, GSK591_2110, Gallibiscoquinazole_1830, PRIMA-1MET_1131 and JAK1_8709_1718, the treatment sensitivity of patients in the low-risk group of the lysosomal pathway is significantly higher than that of the high-risk group of the lysosomal pathway; while for Entinostat_1593, its therapeutic sensitivity increased with higher lysosomal-risk score, which depends on further studies to confirm.

### Cellular validation of MT1X, a key lysosomal pathway gene, on ESCC invasion and physiological reproduction

**Figure 7:**
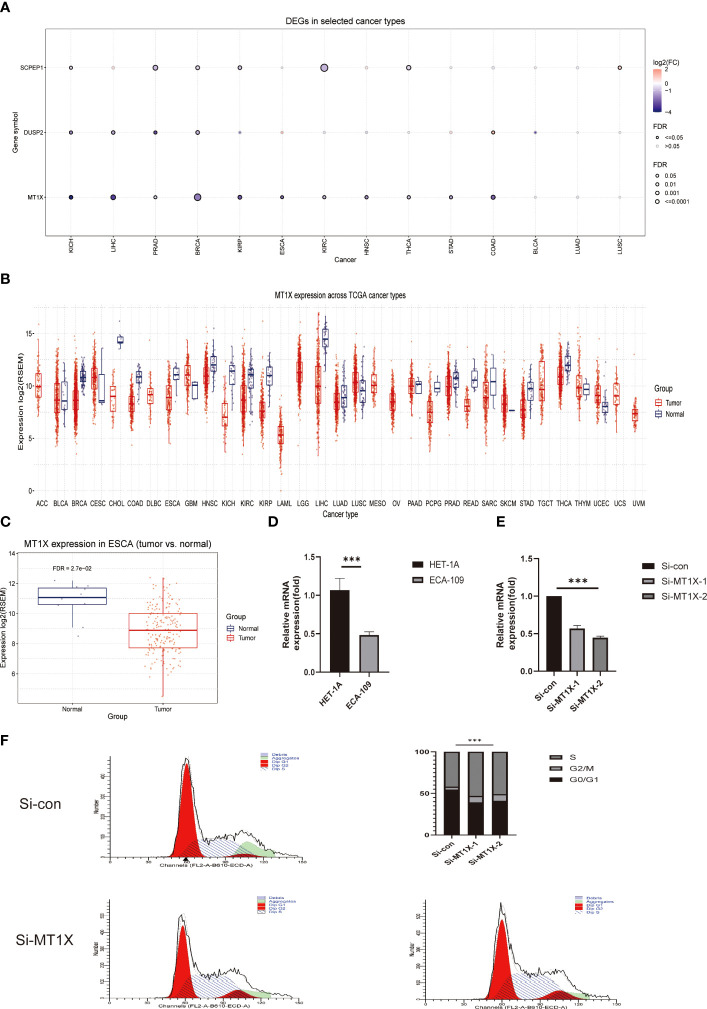
Cellular experimental validation of the role of MT1X, a key gene of the lysosomal pathway, in the growth and invasion of ESCC. **(A)** Box plot analysis reveals the number and significance of the four constitutive genes of the lysosomal pathway risk model in different tumours; **(B)** Box plot analysis of MT1X expression in different tumour types and paraneoplastic tissues, showing that MT1X gene expression was weaker in most tumour types than in paraneoplastic tissues, except for CESC, GBM, UCEC and LUSC **(C)** Box plot analysis of MT1X expression in tumour and normal tissues in ESCC, showing that MT1X expression in normal tissues was significantly higher than that in tumour tissues; **(D)** Cellular assays demonstrating the role of MT1X in the expression and growth invasion of oesophageal cancer cells: qPCR analysis of MT1X expression in ECA-109 and HET-1A cell lines; **(E)** qPCR analysis of MT1X expression in ECA-109 and HET-1A cell lines. qPCR confirmed the successful knockdown of MT1X in ECA-109 cells in two replicate sets; **(F)** Flow cytometry analysis of the cell growth cycle percentage of ECA-109 cell lines before and after MT1X knockdown. *** means <0.001.

Following the above single-cell sequencing and transcriptomic studies and bioinformatic validation, we have initially demonstrated that a risk model consisting of four lysosomal pathway-related genes is strongly associated with immune infiltration and chemo-sensitivity in ESCC. Therefore, we wanted to further validate this organic association experimentally. Here, we selected the MT1X gene, which has the highest weight in the risk model, and performed corresponding knockdown and suppression experiments in oesophageal cancer cells and normal cells to assess its effect on the development of ESCC. First, we analysed the number and significance relationships of several constitutive genes in the lysosomal pathway risk model in different carcinomas (**Figure 7A**). The results showed that the SCPEP1 gene was highly expressed in BRCA, KIRC and THCA tumour types, and the DUSP2 gene was more expressed in KICH, LIHC and BRCA. In contrast, for the MT1X gene, his expression was more significant in BRCA, while for ESCC, its expression level was more limited. To explore the role played by MT1X, the most critical gene in the lysosomal pathway, in ESCC, we further analysed the expression of MT1X in different tumour types using box-line plots in **Figure 7B**. The results showed that MT1X gene expression was weaker in tumour cells than in paraneoplastic tissues in most tumour types, except CESC, GBM, UCEC and LUSC. Further, we analysed the expression of MT1X in tumour and normal tissues in ESCC in **Figure 7C**, which showed that MT1X expression was significantly higher in normal tissues than in tumour tissues. MT1X expression was determined by flow cytometry in esophageal cancer cells ECA-109 as well as in normal esophageal epithelial cells HET-1A (**Figure 7D**), again in agreement with the above results, i.e. the expression of MT1X was lower in esophageal cancer cells. Correspondingly, to clarify the role of key genes of the lysosomal pathway in the development of ESCC. We knocked down MT1X gene expression in ECA-109 cells, and the success of the knockdown is shown in the qPCR results in **Figure 7E**, where MT1X gene expression was significantly lower in both knockdown cell groups. In **Figure 7F**, we analysed the cell cycle distribution for tumour cells as well as for ECA-109 cells after MT1X knockdown using flow cytometry. The results show that when the MT1X gene was further knocked down in the esophageal cancer cells, the number and proportion of tumour cells in the S and G2+M phases of the cell cycle increased. This suggests that the proliferation and growth of esophageal cancer cells were significantly promoted after knocking down the MT1X gene. It is suggested that the MT1X gene in the lysosomal pathway may be associated with the proliferation and growth of tumour cells.

## Discussions

As a common malignant tumour of the digestive system, oesophageal cancer still has a poor overall survival rate and a poor prognosis, although its incidence and mortality rate have been significantly reduced in recent years (14, 15). The lysosomal pathway, as an important underlying mechanism in tumour cell metabolism, invasion, metastasis and development, is inevitably associated with poor immunotherapy outcomes and poor prognosis in ESCC patients (16, 17). The autophagic lysosome system is a cellular degradation system that plays an important role in the regulation of proteins, lipids and cell homeostasis. Therefore, the autophagic lysosome system can play key functions in a variety of diseases, including cancer, immune and inflammation-related diseases, etc (18). It has been found that lysosomes regulate the growth and proliferation of tumor cells by activating the growth factor signaling pathway through tyrosine kinase receptors on the membrane. the uncontrolled proliferation of tumor cells often requires more nutrients to maintain cell metabolism. Lysosomes can degrade proteins inside and outside cells through autophagy and pinocytosis, and provide a large amount of amino acids to cells (19). Therefore, the relationship between lysosomes and cancer is inextricably linked. LAMP1 is the main protein component located on the lysosome membrane. Immunohistochemical staining of ESCC patients indicated that LAMP1 expression level was significantly different between TNM stage and tumor histological differentiation degree. This also indicates that lysosomes are closely related to the occurrence and development of ESCC (20). The study of the lysosome pathway related to the development of ESCC and immune infiltration is of great significance and has a guiding role in the development of new targeted therapy strategies for ESCC.

In this study, we identified genes related to the lysosomal pathway based on the GEO database, and systematically identified the relevant biological pathways and pharmacological sensitivities of these genes. Single-cell sequencing was used to analyse the close association between lysosomal pathway-related genes and the gene distribution of oesophageal cancer. Pathway enrichment analysis revealed that lysosomal DEGs were significantly associated with various elements of tumour progression, such as metabolism, cellular processes and biological systems. We found that the ECM-receptor interaction pathway was enriched. Extracellular matrix (ECM) is an important component of the tumor microenvironment. It has various functions, including mechanical support and regulation of the microenvironment. In the process of tumorigenesis, the interaction between cancer cells and tumor microenvironment (TME) often leads to the stiffness of ECM, thus causing further tumor deterioration (21). Thus, ECM plays an important role in tumor progression. Cytokines are the key proteins of signaling in the tumor microenvironment (TME) and have pleiotropy. It can be divided into interleukin, interferon, tumor necrosis factor, hematopoietic factor, growth factor, chemokine receptor interaction and so on. Cytokine-cytokine receptor interaction plays an important role in the occurrence and development of tumor. Interferon and TGF-β can directly or indirectly inhibit tumor cell growth, TNF and various chemokines play a role by promoting angiogenesis, and IL-18 can activate NF-κB signal, induce cancer cell proliferation and invasion, and prevent cell apoptosis (22). All the mechanisms mentioned above indicate the close relationship between cytokines and tumors. In addition, IL-17 signaling pathway was also enriched. According to the study of Chen et al., IL-17 can promote the recruitment and activation of neutrophils in esophageal squamous cell carcinoma, thus playing a role in anti-tumor immunity (23). Meanwhile, IL-17 is also a kind of cytokine, which indicates that lysosome-DEGs is closely related to cytokines. The lysosomal pathway prognostic risk model is a significant predictor of prognosis in ESCC patients. It also highly influences the immune infiltration and chemosensitivity of ESCC patients. On this basis, we further confirmed the microscopic role of the lysosomal pathway in the invasion and metastasis of esophageal cancer by knocking down the lysosomal pathway gene - MT1X in cellular assays. This suggests that the lysosomal pathway and its related genes play an important role in the development of ESCC and drug resistance, and the study of the organic interaction between esophageal cancer-lysosomal pathway-immune infiltration may become a breakthrough for further exploration of esophageal cancer.

The common treatment option for oesophageal cancer is surgical resection followed by chemotherapy and radiotherapy, but patients are more prone to develop resistance, which affects the therapeutic effect. The lysosomal pathway promotes the establishment of drug resistance in cancer cells (18). Currently, the commonly used clinical inhibitors of the lysosomal pathway are chloroquine and its derivative chloroquine, an antimalarial drug that prevents lysosomal acidification and prevents lysosomal pathway vesicles from being cleared (19). Clinical studies (20, 21) have demonstrated the therapeutic potential of chloroquine and its derivative hydroxychloroquine, alone or in combination with other drugs, to improve the efficacy of radiotherapy in patients with melanoma, colorectal cancer, myeloma and renal cell carcinoma by inhibiting the lysosomal pathway. However, no studies have been conducted using these lysosomal pathway inhibitors in patients with ESCC. The lysosomal pathway plays an important and complex regulatory role in the development and progression of oesophageal cancer and can influence its therapeutic outcome. Although the lysosomal pathway has been shown to play a protective role in the conversion of BE to EAC, further *in vivo* models need to be developed to investigate the regulatory mechanisms of the lysosomal pathway. When considering the use of lysosomal pathway inhibitors in the treatment of oesophageal cancer, it is critical to understand whether the cell body and the underlying lysosomal pathway levels are being disrupted. It is important to accurately determine whether a patient’s lysosomal pathway is activated or deactivated, and to combine this with factors such as whether they are receiving radiotherapy. In addition, due to the complexity of lysosomal pathway regulation in tumours, how to judge the level of basal autophagy and assess the role played by autophagy still requires further development of new assay systems to achieve specific regulation of lysosomal pathway levels and better guide clinical treatment. This study attempts to make some breakthroughs in this field and to identify important genes related to the lysosomal pathway. On the basis of this, risk models will be constructed and direct associations between them and immune infiltration will also be investigated. This may advance the process of lysosomal targeting and therapeutic resistance research in ESCC.

This study also has some limitations. Firstly, as a retrospective analysis, the data obtained were mostly from public databases, and although we have performed a preliminary validation through cellular experiments, there is a need for rich mechanistic studies in the future. As for the key genes that were previously screened, we were able to find downstream molecules through mass spectrometry and proteomics analysis. It was verified by molecular biology experiments. In addition, we can further verify this *in vivo* using knockout mice. Secondly, the study set lacks important molecular and clinical data on ESCC patients, and these pathological factors, which are more relevant to clinical treatment decisions, may also be associated with lysosomal pathway actions. Also, the GEO database lacks complete treatment records, such as chemotherapy regimen selection or targeted therapy information. We can use clinical samples from our hospital to further explore the correlation between lysosome pathway molecules and cancer through immunohistochemical and clinical data analysis, as well as immunotherapy analysis. It is hoped that future studies will include more molecular pathology and clinical information, and that basic research will be used to analyse the interaction between MT1X and ESCC targets in depth, in order to more fully explore the close association between the lysosomal pathway and ESCC. We can further explore this with prospective studies.

Herein, based on single cell sequencing and transcriptomic analysis, we investigated the microscopic role of the lysosomal pathway and related genes in the development of ESCC, and confirmed that there is a close association between the lysosomal pathway and the immune infiltration and immune pathway of ESCC. The preliminary validation was performed by cellular assay. This suggests that studying the interaction between the lysosomal pathway and immune infiltration and immune cells may be a potential target to promote new directions in the treatment of ESCC.

## Data availability statement

Publicly available datasets were analyzed in this study. This data can be found here: MSigDB database (GSE53624, GSE188900).

## Author contributions

JW was responsible for the main article conception, data collection and processing. XG was responsible for full-text operation, testing the validity of articles. The rest were responsible for assisting the above two to improve the content of the article.
