# Supplementary material for: Spatial heterogeneity and Immune infiltration of cellular lysosomal pathways reveals a new blueprint for tumor heterogeneity in esophageal cancer
Source: Front Endocrinol (Lausanne). 2023 Apr 5;14:1138457. doi: 10.3389/fendo.2023.1138457 (PMC10113631; doi:10.3389/fendo.2023.1138457)

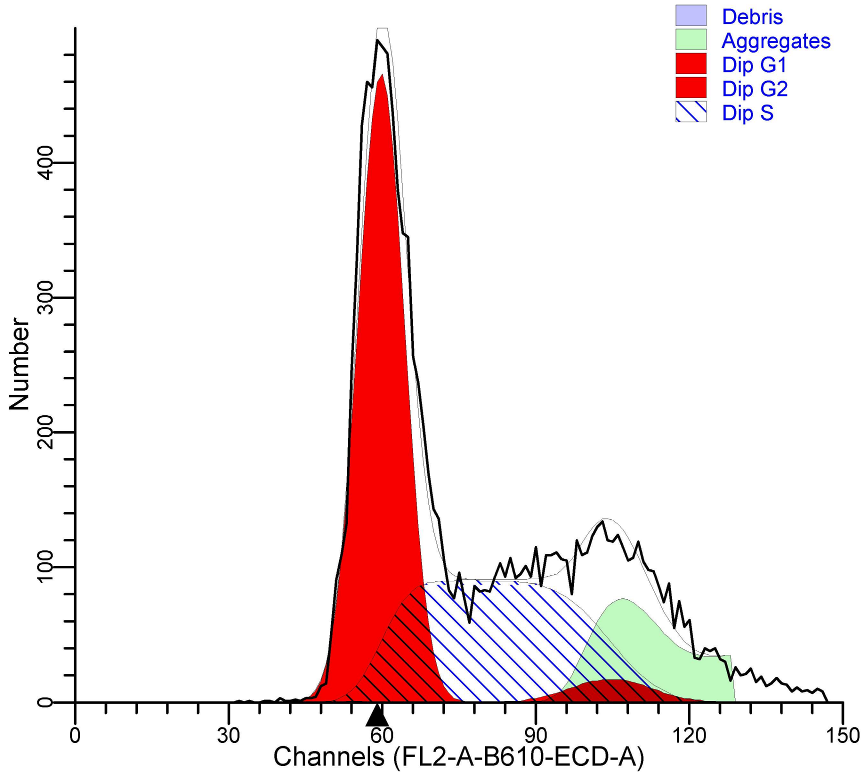

File analyzed: c on-1.fcs
Date analyzed: 11-Nov-2022
Model: 1DA0n_DSD
Analysis type: Manual analysis

Ploidy Mode: First cycle is diploid

Diploid: 100.00 %
Dip G1: 54.69 % at 59.76
Dip G2: 3.55 % at 104.59
Dip S: 41.76 %   G2/G1: 1.75
%CV: 7.59

Total S-Phase: 41.76 %
Total B.A.D.: 5.61 %

Debris: 0.22 %
Aggregates: 14.48 %
Modeled events: 11393
All cycle events: 9719
Cycle events per channel: 212
RCS: 3.466

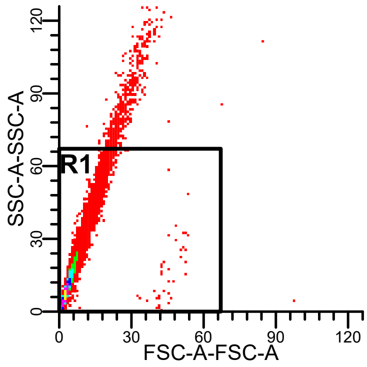

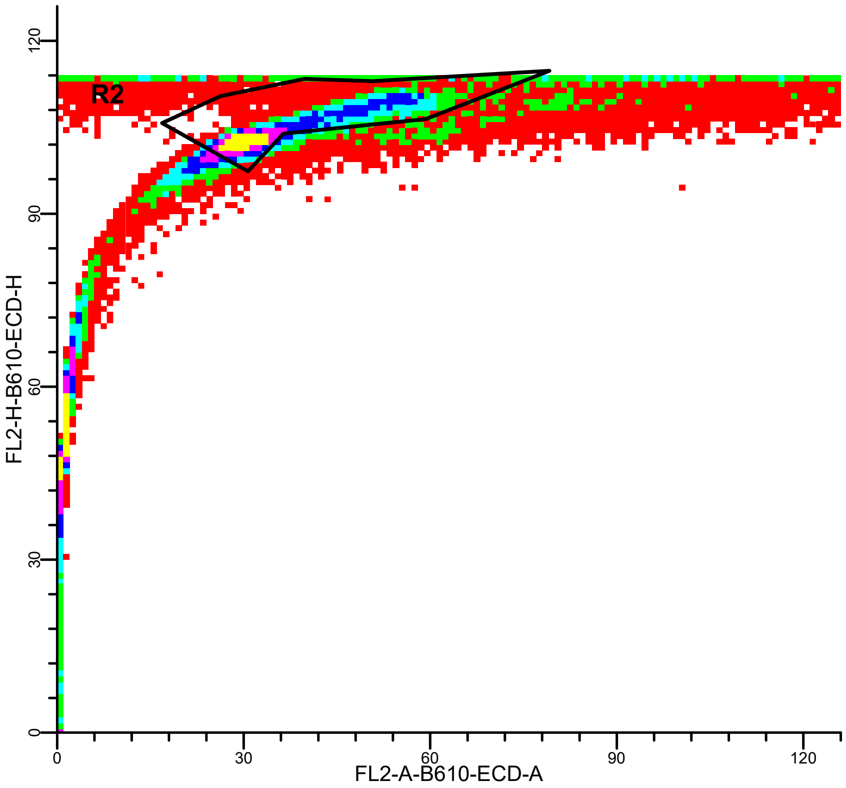

Supplement: Supplementary file 1 [file DataSheet_1.zip › experimental_raw_data/flow cytometry/con-1.pdf]
