# Supplementary material for: Spatial heterogeneity and Immune infiltration of cellular lysosomal pathways reveals a new blueprint for tumor heterogeneity in esophageal cancer
Source: Front Endocrinol (Lausanne). 2023 Apr 5;14:1138457. doi: 10.3389/fendo.2023.1138457 (PMC10113631; doi:10.3389/fendo.2023.1138457)
Supplement: Supplementary file 1 [file DataSheet_1.zip › experimental_raw_data/flow cytometry/si-MT1X-1.pdf]

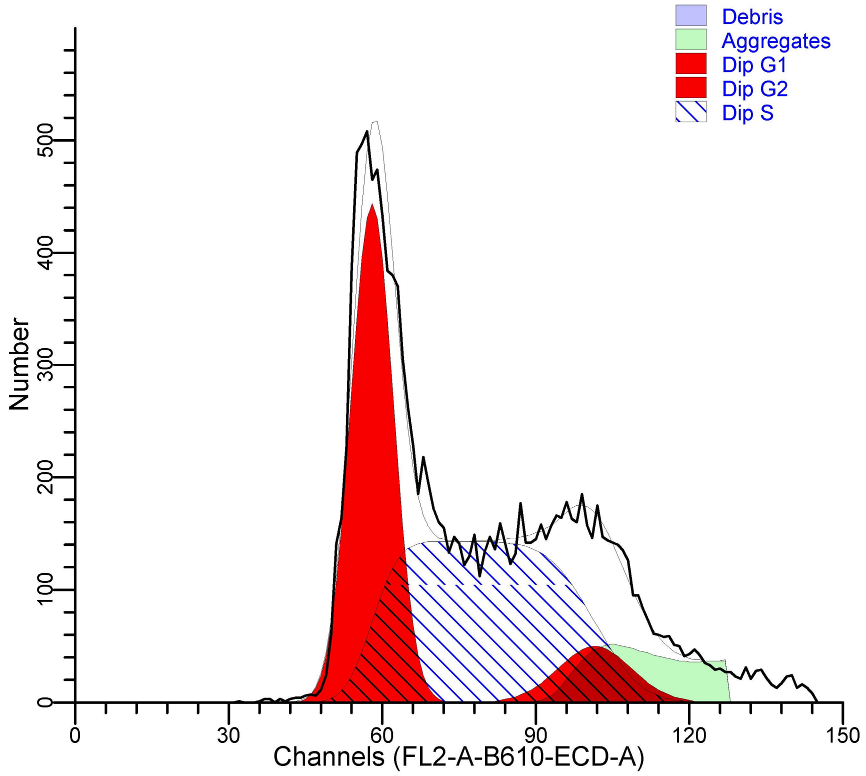

File analyzed: 实验1-1.fcs
Date analyzed: 11-Nov-2022
Model: 1DA0n_DSD
Analysis type: Manual analysis

Ploidy Mode: First cycle is diploid

Diploid: 100.00 %
Dip G1: 39.28 % at 57.99
Dip G2: 7.71 % at 101.49
Dip S: 53.01 %   G2/G1: 1.75
%CV: 7.13

Total S-Phase: 53.01 %
Total B.A.D.: 2.69 %

Debris: 0.20 %
Aggregates: 10.28 %
Modeled events: 13117
All cycle events: 11742
Cycle events per channel: 264
RCS: 4.186

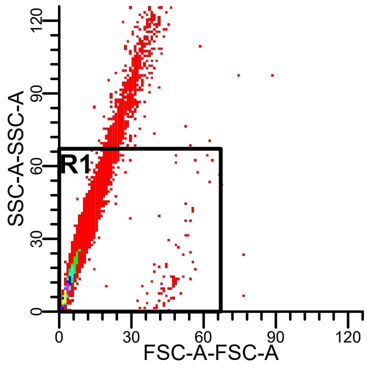

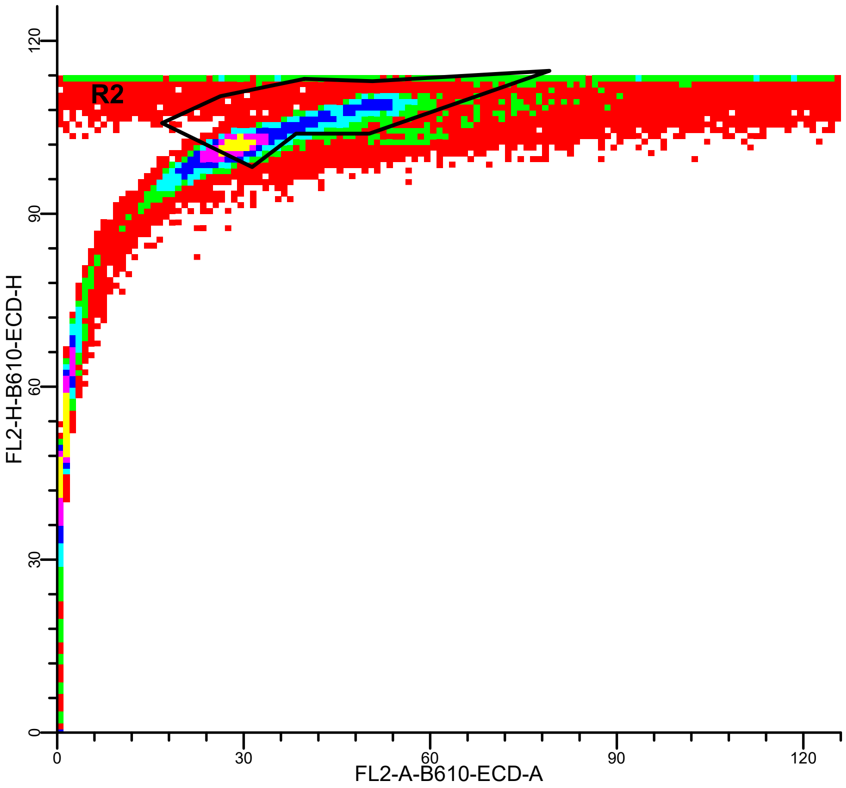

ModFitLT V3.1(Win32)
