# Supplementary material for: Spatial heterogeneity and Immune infiltration of cellular lysosomal pathways reveals a new blueprint for tumor heterogeneity in esophageal cancer
Source: Front Endocrinol (Lausanne). 2023 Apr 5;14:1138457. doi: 10.3389/fendo.2023.1138457 (PMC10113631; doi:10.3389/fendo.2023.1138457)

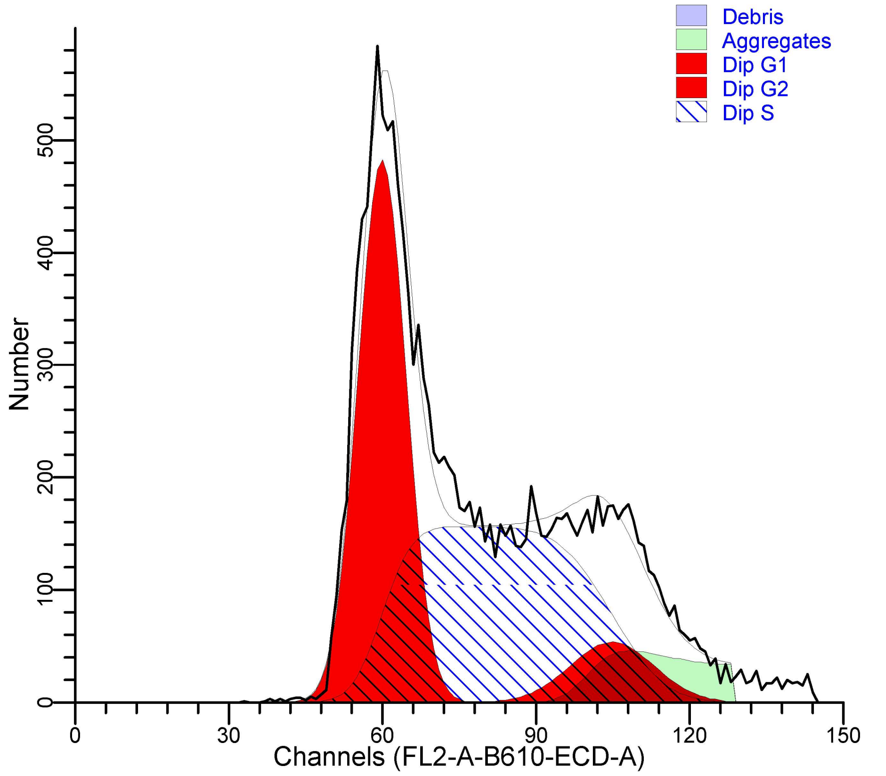

File analyzed: 实验1-2.fcs
Date analyzed: 11-Nov-2022
Model: 1DA0n_DSD
Analysis type: Manual analysis

Ploidy Mode: First cycle is diploid

Diploid: 100.00 %
Dip G1: 41.15 % at 59.89
Dip G2: 7.99 % at 104.81
Dip S: 50.86 % G2/G1: 1.75
%CV: 7.83

Total S-Phase: 50.86 %
Total B.A.D.: 2.30 %

Debris: 0.10 %
Aggregates: 7.95 %
Modeled events: 15017
All cycle events: 13808
Cycle events per channel: 301
RCS: 4.618

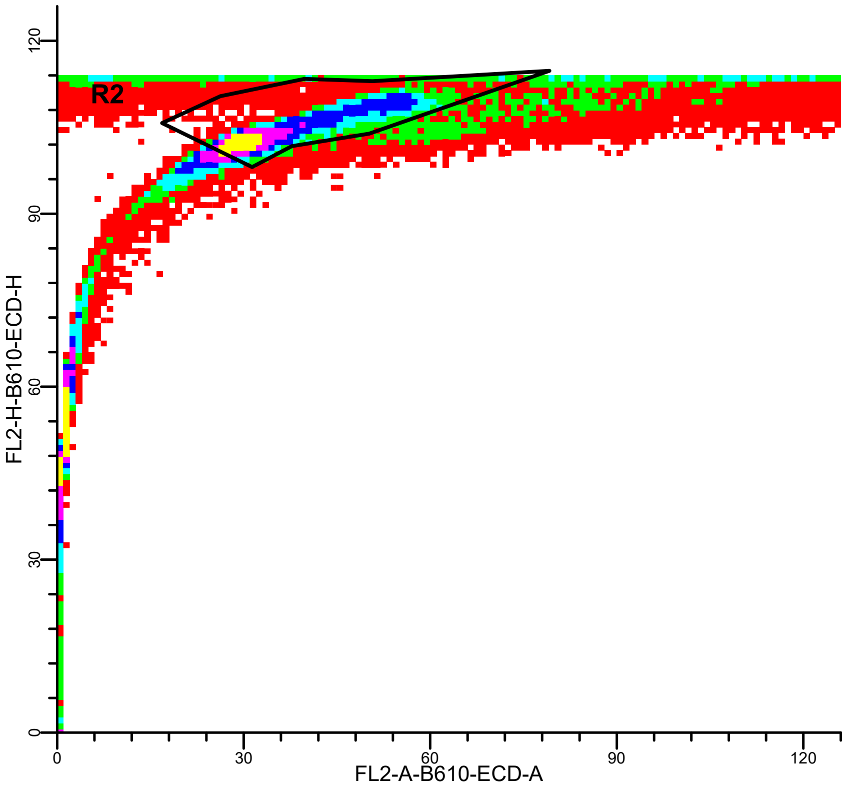

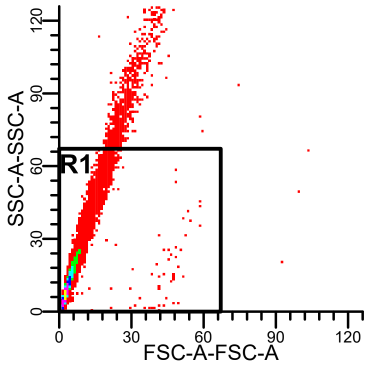

Supplement: Supplementary file 1 [file DataSheet_1.zip › experimental_raw_data/flow cytometry/si-MT1X-2.pdf]
