# Supplementary figures and images for: Spatial heterogeneity and Immune infiltration of cellular lysosomal pathways reveals a new blueprint for tumor heterogeneity in esophageal cancer
Source: Front Endocrinol (Lausanne). 2023 Apr 5;14:1138457. doi: 10.3389/fendo.2023.1138457 (PMC10113631; doi:10.3389/fendo.2023.1138457)

\*\*\*

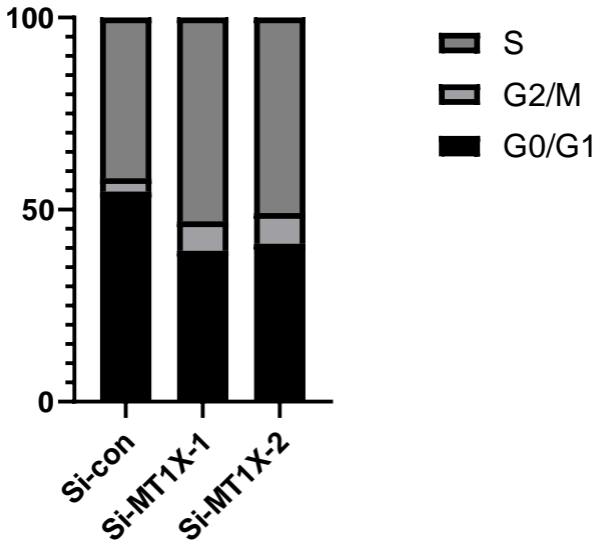

Supplement: Supplementary file 1 [file DataSheet_1.zip › experimental_raw_data/flow cytometry/Data 1.pdf]
