# Supplementary material for: Spatial heterogeneity and Immune infiltration of cellular lysosomal pathways reveals a new blueprint for tumor heterogeneity in esophageal cancer
Source: Front Endocrinol (Lausanne). 2023 Apr 5;14:1138457. doi: 10.3389/fendo.2023.1138457 (PMC10113631; doi:10.3389/fendo.2023.1138457)
Supplement: Supplementary file 2 [file Image_1.pdf]

Supplementary Information

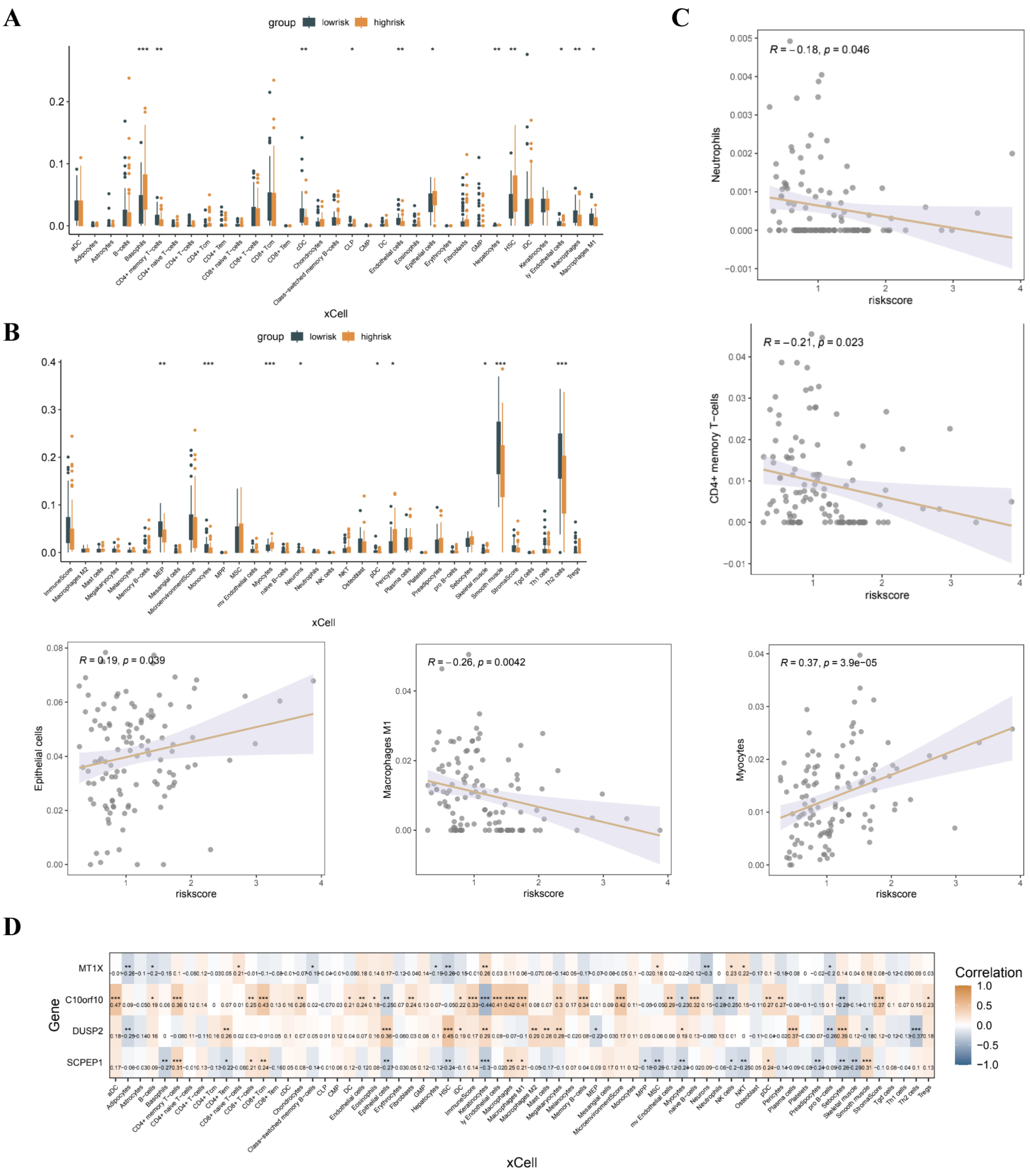

Figure S1. Immune infiltration assessment of lysosomal pathway risk scores obtained by XCell in ESCC patients

A. Box line plot of immune cell scores calculated by XCell method between low risk and high risk groups; CD4 Memory T cells, Macrophages, and Marcophages M1 expression were higher in the low risk group than in the

high risk group; while Basophils, CLP, Epithelial, and HSC cells were lower in the low risk group than in the high risk group; B. Box plot of immune pathway expression scores between the low-risk and high-risk groups calculated by the XCell method; C. Linear correlation between several immune cell types with significantly different expression between the low-risk and high-risk groups and their respective risk score expression, including Neutrophils, CD4 memory T cells, Macrophages M1, Epithelial cells, and Myocytes.D. Correlation heat map analysis of genes constituting a risk model for the lysosomal pathway (including MT1X, C10orf10, DUSP2, SCPEP1) interacting with immune infiltrating cells and pathways in the XCell method.

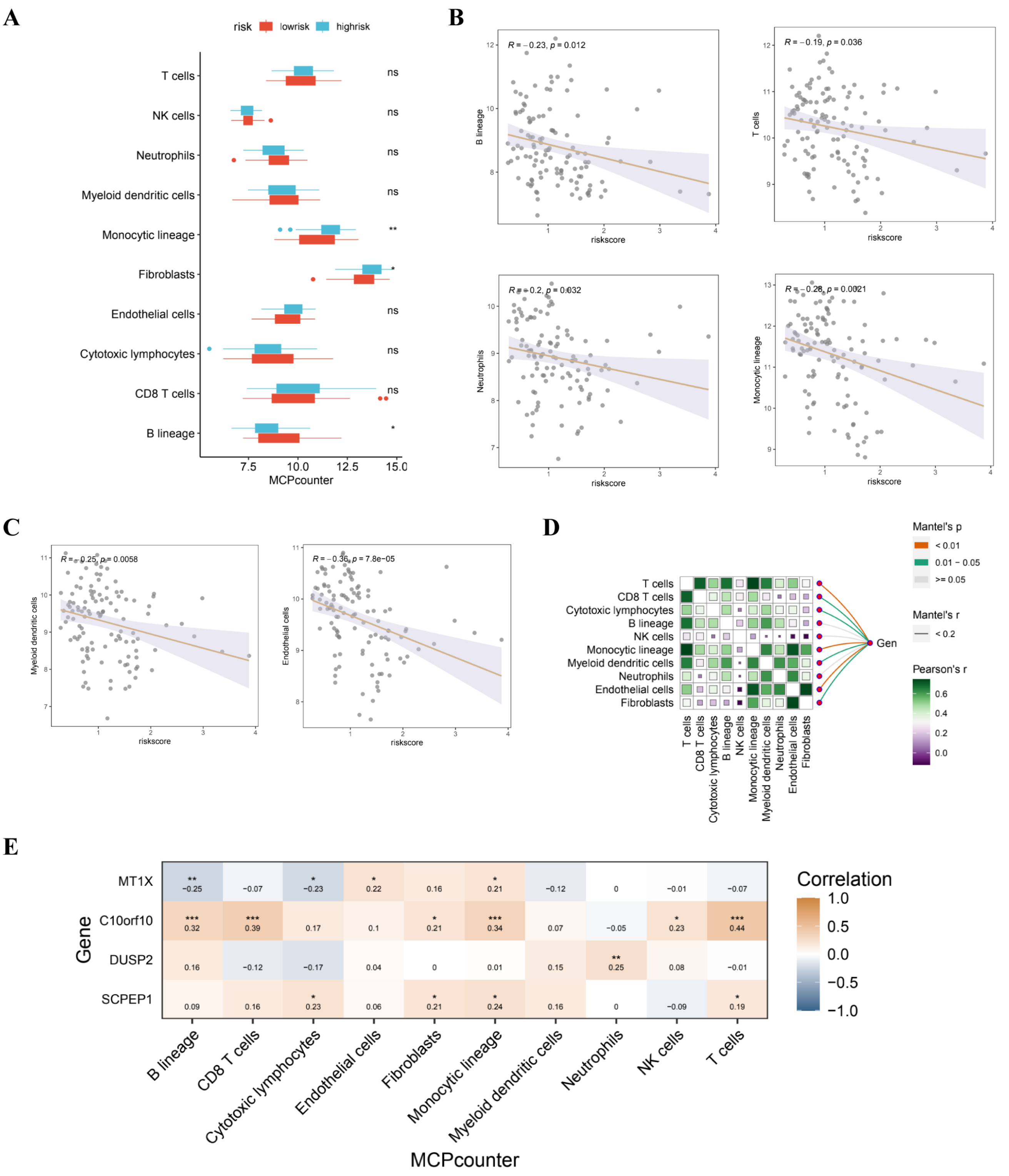

Figure S2. Immune infiltration assessment of lysosomal pathway risk scores obtained by the MCP method in ESCC patients

A. Box line plot of immune cell scores calculated by the MCP method between the low and high risk groups of the lysosomal pathway; the expression of Fibroblasts was lower in the low risk group than in the high risk

group, while the expression of B lineage was higher in the low risk group than in the high risk group; B. The immune cells of B lineage, T cells, Neutropilis, and Monocytic Lineage were correlated with B lineage, T cells, Neutropilis, and Monocytic Lineage with the expression of their respective risk scores; C. Linear correlation of Myeloid dendritic as well as Endothelial cells with the expression of their respective risk scores; D. Heat map using Pearson correlation to reveal the correlation of expression of different immune cells, immune pathways; E.A. Correlation heat map analysis of genes that constitute the risk model of lysosomal pathway (including MT1X, C10orf10, DUSP2, SCPEP1) interacting with immune infiltrating cells and pathways in the MCP method.
